# Effects of a Novel Dental Gel on Enamel Surface Recovery from Acid Challenge

**DOI:** 10.4172/2161-1122.1000397

**Published:** 2016-10-25

**Authors:** Tracie Lam, Jessica Ho, Afarin Golabgir Anbarani, Lih-Huei Liaw, Thair Takesh, Petra Wilder-Smith

**Affiliations:** Beckman Laser Institute, University of California, Irvine, CA, USA

**Keywords:** Erosion, De-mineralization, Re-mineralization, Scanning Electron Microscopy (SEM), Dental gel, Dentifrice

## Abstract

**Background:**

Objective was to evaluate the *in vivo* effects of a novel dental gel (Livionex gel^R^) vs. a comparison dental gel on the surfaces of pre-eroded enamel chips.

**Methods:**

On days 1–5, after toothbrushing with dentifrice, nine subjects each wore 8 enamel chips mounted on a palatal appliance for 4 h. Enamel blocks were pre-demineralized daily. After 2 day washout, subjects repeated the protocol using fresh chips and the second toothpaste on days 8–12. Samples were evaluated using electron microscopy.

**Results:**

Ten standardized enamel surface photomicrographs/sample (total 1440 images) were evaluated for signs of erosion visually and on a scale of 0–3 by 1 evaluator. No significant differences were found between the 2 groups (p>0.32, 95% C.I.). Minimal surface erosion on approx. 15% of sample area was visible in both groups.

**Conclusion:**

The enamel surface appeared similar after usage of a test or control dentifrice. Based on this study, the test formulation did not affect enamel surface recovery from an erosive challenge.

**Practical implications:**

Dentifrices can contribute to maintaining a healthy enamel surface. An all-natural dental gel formulation with novel anti-plaque mechanism achieved similar recovery from acid challenge to enamel as a control gel.

## Introduction

Dental erosion develops from chronic exposure to non-bacterial acids, resulting in mineral loss from the tooth surface and reduced surface micro-hardness. Clinical manifestations such as shallow lesions on smooth surfaces and cupping and flattening of cusps can develop even in early stages, which can lead to the exposure of coronal dentine. Dental hypersensitivity is common in patients with erosion, and in the long-term progressive loss of tooth substance can become so extreme that tooth fracture may ensue. Causes of erosion include inappropriate oral hygiene regimens, gastric reflux, unusual dietary patterns and the consumption of acidic foods and beverages [1]. In the United States alone, soft drink consumption has increased by 300% in the last 20 years [2]. Although prevalence data is not homogenous, there exists a trend in recent years towards more pronounced rates of dental erosion even in younger age groups [3], attributed in part to the substantial replacement of milk with soft drinks [4]. The intake of dairy products can have hardening effects on dental enamel [5]. In mild cases of enamel softening, dairy product consumption can assist enamel hardening or re-mineralization, and this observation is attributed to the calcium and phosphate provided by these products as well as an increased rate of salivary flow that can be associated with dairy consumption [5,6]. Thus the consumption of a demineralizing soft drink as a replacement for a remineralizing beverage can produce a “double negative” effect on dental health. Components of a “healthy” diet can also provide considerable acidic challenge to the teeth, including fruit, fruit juices, sparkling fruit drinks and even salad dressings as well as coffee and wine.

During the initial stages of enamel erosion, demineralization is paralleled by reduced enamel surface hardness [7–9], resulting in heightened risk of abrasion and attrition [10–12]. The rate of demineralization depends on various factors including the pH and duration of the acid challenge [9–15]. Prior to actual tissue loss, remineralization can occur through the replacement of lost mineral ions from the salivary reservoir of calcium and phosphate ions [9–16]. Dentifrices, especially those containing fluoride formulations, can be helpful in supporting dental remineralization by increasing the acid resistance of tooth surfaces or pellicles and/or promoting remineralization after acid attack [1,2,17] However, because one of the primary functions of toothpastes is to remove plaque, many dentifrice formulations also contain abrasives, which, although otherwise beneficial in terms of cleaning properties, may counteract the product’s potential role in preventing demineralization and/or promoting remineralization through physical abrasion of the softened tooth surface [17]. Once erosion has progressed to actual tissue loss, it can no longer be reversed and must be treated with restorative therapies such as tissue replacement by dental resins or cements. More severe erosive lesions may require treatment with more extensive measures, such as ceramic veneers, overlays, and crowns. These procedures are expensive and provide a measurable amount of discomfort to the patient [18]. Thus the clinical importance of erosion-preventative measures is considerable.

Toothpastes are mostly complex formulations consisting of multiple active ingredients that target a range of desired effects. Their action in the frame of dental erosion is less well investigated and understood than in caries prevention, where active ingredients primarily target subsurface and approximal surface sites that are sheltered from direct physical trauma. Erosion, however, primarily occurs on plaque-free smooth surfaces and the occlusal areas, where specific active dentifrice ingredients may offer protection, while abrasive components also contained in the dentifrice can be a counteracting factor. The interplay of both is not fully elucidated.

The objective of this study was to evaluate the *in vivo* effects of a novel dental gel (Livionex gel^R^, Los Gatos, CA) vs. a control dentifrice on enamel microstructure after repeated erosive challenges. Unlike the control dental gel the test dental gel contains no fluoride, triclosan, detergents or abrasives.

## Materials and Methods

### Protocol overview

This exploratory study was designed as a single center, blinded dental examiner, subject and laboratory analyst, crossover treatment regimen. An *in situ* model using 8 enamel chips per retainer in 9 subjects over 2 cycles (total of 144 enamel chips) was used to evaluate enamel surface response to a cycle of *ex vivo* erosive challenge and *in vivo* dental gel use followed by 4 h exposure to the oral environment. This cycle was repeated over 5 days, followed by a 2-day washout period, and then a crossover to use of the second dental gel for 5 days. Finally, enamel samples were imaged with Scanning Electron Microscopy (SEM) to visualize surface microstructure, which was evaluated in 3 low-resolution images (for orientation) and 10 high-resolution (x1000) photomicrographs (for scoring) per sample. One blinded, experienced, pre-standardized scorer evaluated all 1440 high-resolution images on a scale of 0–3 for surface changes. This research was performed in full compliance with the University of California at Irvine’s (UCI) IRB-approved protocol #2013-9778.

### Subject selection

Nine subjects ranging in age from 19–54 years old (mean age of 37 years) were enrolled in this prospective, randomized, double-blinded crossover study. Subjects were recruited by e-mail. Sample size was calculated based on data from a prior pilot study. 6 subjects were female and 3 were male; 5 were Caucasian and 4 Asian. Subjects were screened to exclude persons with any known history of allergy to personal care/consumer products or their ingredients, and any ingredients in the test product. Other exclusion criteria included GERD, any medical condition which requires pre-medication prior to dental visits/procedures, any diseases of the soft or hard oral tissues including a gingival index, plaque index or SBI >2, use of antibiotics within one month of study begin, pregnancy or lactation, as well as immune compromised individuals (HIV, AIDS, immuno-suppressive drug therapy). The participants were randomized in 1 group of 9 with regard to sequence of dentifrice use.

### Enamel chip samples

**Figure 1:**
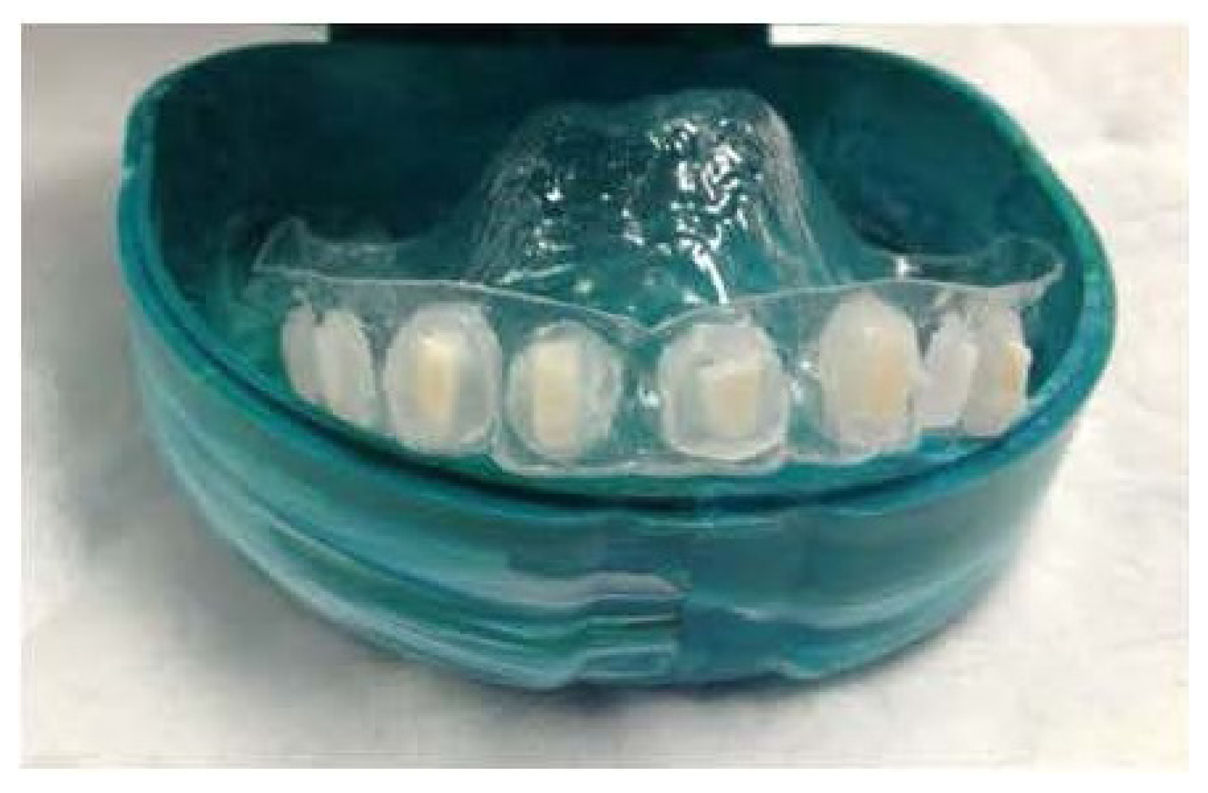
Oral appliance.

One hundred and forty-four sterilized enamel chips were subjected to an *ex vivo* erosive challenge by individual exposure to 50 mL of grapefruit juice with a mean pH of 3.42 ± 0.05 and mean titratable acidity of 181 ± 8 mmol of hydroxide ion per liter of juice. After 25 min exposure to the grapefruit juice at room temperature, samples were rinsed with deionized water for 2 min, and then mounted onto a custom-fabricated removable intra-oral appliance with sticky wax (Figure 1) [15].

### *In vivo* protocol

Subjects employed a fluoride free washout dentifrice (Tom’s of Maine, Kennebunk, ME 04043) for two days prior to the study, and again between legs 1 and 2 of the study. They were randomized as to sequence of dentifrice use. Four subjects used the test dental gel first (Livionex^R^, Los Gatos, CA), and the remaining 5 subjects used the control dental gel (Colgate Total^R^, Colgate-Palmolive, New York, NY) in the first leg of the study. On the first day of leg 1, under clinical supervision, subjects brushed and flossed all of their teeth using standardized technique with the allocated dental gel. Then they brushed the buccal surfaces of their maxillary teeth with 1.5 g of the allocated dental gel for 30 s and, without expectorating the slurry, they then placed the appliance in their mouth and rinsed the slurry around the palatal appliance for 60 s. Neither the appliance nor enamel specimens were brushed. Following expectoration subjects rinsed gently with tap water (15 mL, 10 s) before again expectorating. After 4 h the appliance was removed from the mouth and stored (4°C, 100% humidity). Eating was prohibited whilst wearing the appliance; however, drinking up to 2 cups of water was permitted after the first hour. This process was repeated in the subjects’ homes twice daily for 5 days for each leg of the study.

### SEM imaging

After sample removal from the appliance on day 5 of each leg of the study, specimens were dehydrated in a graded series of aqueous ethanol (50, 70, 90, and 100% ethanol) for 10 min at each concentration. Then, they were mounted on stubs using colloidal silver liquid (Ted Pella, CA, USA), and gold coated on a PAC-1 Pelco advanced coater 9500 (Ted Pella, CA, USA). Photomicrographs of the enamel surface were recorded by a blinded technician utilizing a Philips 515 (Mohawk, NJ, USA) scanning electron microscope. Three low-resolution photographs for the purposes of orientation, and 10 high-resolution (x5000) photomicrographs were recorded per sample. All 1440 images were evaluated by 1 blinded, experienced, pre-standardized scorer on a scale of 0–3 for surface changes. Scores were allocated as follows:

0 Visible demineralization on 0–10% of photomicrograph surface;
1 Visible demineralization on 11–40% of photomicrograph surface;
2 Visible demineralization on 41–70% of photomicrograph surface;
3 Visible demineralization on 71–100% of photomicrograph surface.

## Results

**Figure 2:**
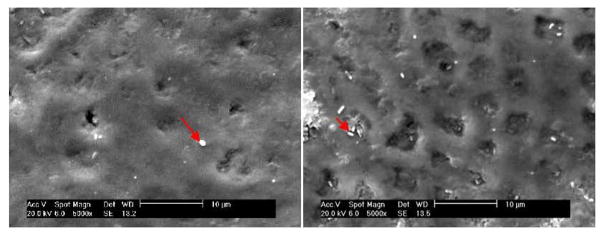
Representative SEM photomicrographs (x5000) of specimens after completion of the experimental protocol using the control dental gel. The enamel surface layer is mainly intact. Arrows indicate bacteria on the simple surface.

Samples appeared unaltered to the naked eye at the culmination of this study. In both groups, SEM images showed some small areas of enamel defects or loss as well as altered surface appearance of roughness (Figure 2). Mean surface score for the test group was 1.6 (0.2) and for the control group it measured 1.5 (0.2). In the samples exposed to the control dental gel, the altered patches of enamel appeared mildly cratered with some roughness and pitting (Figure 2). The surfaces of samples exposed to the test dental gel demonstrated a somewhat more homogeneous appearance, with localized changes presenting as smooth, shallow saucer-like defects (Figure 2). Enamel surface scores were computed for each sample and formed the basis of the comparison between the two dentifrices. Sums and differences of the scores between study legs were calculated for each subject. Means and standard deviations were calculated for each dentifrice group based on dentifrice sequence of use, where group 1 included subjects who used the test gel first, and group 2 consisted of subjects who had brushed first with the control gel. The sums and differences were tested for significance by means of a t-statistic. The t-test was performed on the sums of the differences to determine whether there was a carryover effect in any of the indices. The sequence of gel use did not show any significance in the test (p>0.44, 95% C.I.). A t-test was also performed to elucidate whether one treatment resulted in a different enamel surface score than the other treatment. No significant differences were found between the 2 groups (p>0.32, 95% C.I.).

## Discussion

Dental erosion is a multi-factorial condition wherein an initial softening of the surface in response to an erosive challenge to the enamel is eventually followed by permanent loss of the demineralized tooth structure [19]. Additional factors contributing to the erosive properties of materials entering the oral cavity include their mineral content, ability to complex with calcium, and their buffering capacity, as well as the composition and flow rate of saliva [20]. The degree of saliva and plaque saturation with regard to dental minerals such as hydroxyapatite and fluorapatite also affect outcomes of the erosive challenge [21]. Using SEM analysis many researchers have characterized the dental demineralization and dissolution that result from erosive challenge [22]. On the enamel surface, initial damage occurs to the prism sheath area, followed by dissolution of the prism core, resulting in a honeycomb appearance under high magnification [23]. Further diffusion of acid into the interprismatic area of the enamel results in progressive mineral loss [24].

In the current study, teeth underwent citric acid erosive challenge. In order to ensure comparability with the results of previous studies, an established protocol using grapefruit juice was adopted [25,26]. Orange juice and grapefruit juice alike contain high levels of citric acid that causes considerable erosion [27], and both have been used widely as a simple model of acid erosion. In those studies, SEM micrographs of the tooth surface showed a surface etching effect on the enamel, which is consistent with the early stages of the erosive process [27,28].

Each dental gel used in this study was applied as a slurry that remained in the mouth after brushing the natural teeth. The sample-carrying appliance was inserted immediately into the oral cavity, the slurry was swished around the samples on the retainer for 60 s before expectorating, and then the retainer remained in the mouth for another 4 h daily. A slurry rather than a contact tooth brushing model was chosen to avoid the potentially confounding effects of variables in tooth brushing techniques, and to eliminate any effects of differing levels abrasiveness of the 2 dental gels. While several studies have demonstrated comparable effects of dentifrices on enamel erosion when using a brush vs. a slurry technique, other studies have determined considerable differences between the 2 application techniques [29–32]. Such uncertainties are common in this under-researched field, and they considerably hamper study design and interpretation of research results.

Sound enamel primarily is made up of calcium and phosphate crystallites densely packed in a prismatic structure. The mineral content of enamel is around 87% by volume [4]. During an erosive challenge, if the acidic liquid surrounding the tooth surface is under-saturated with respect to tooth minerals–like the grapefruit juice used in this study–mineral dissolves from the outermost enamel surface and erosive demineralization occurs [2,33]. With continuing erosive challenge, mineral layers are progressively dissolved, causing bulk tissue loss. The partly demineralized residual enamel surface appears etched [5,6]. In cross-section, it appears as a surface less dense band that is a few microns in thickness [7,34]. After an erosive challenge, active ingredients such as fluoride or polyvalent metal cations from a dentifrice or mouthwash interact directly with the eroded enamel surface, whereas in early caries lesions their primary target tissue is at a subsurface location. The partial loss of mineral on the surface is accompanied by a reduction in microhardness, leaving eroded enamel more prone to abrasion and wear [13].

In this study, no significant differences were found in the level of erosion visible in the samples exposed to the test vs. the control gel, despite the fact that the control gel contains fluoride, whereas the test gel does not. It is generally accepted that fluoride plays a major role in caries prevention. However, its role in erosion prevention is less clear [17]. After exposure to fluoride, CaF_2_-like mineral salts are deposited on the tooth surfaces under certain conditions [25,35]; these precipitates are important for protection against caries. However, because they are relatively soluble in acids, they may be less effective in the case of erosion [17]. On this topic the findings in the literature are inconsistent [17]. In a comprehensive review of the literature, the erosion-protective effect of conventional sodium fluoride toothpaste compared to fluoride-free controls was reported to range from “no effect” to 37% protection in enamel [17]. Contradictory results have also been reported for more highly concentrated fluoride formulations. An *in vitro* study comparing a 1,100 and a 5,000 ppm sodium fluoride formulation determined an erosion-protective effect compared to placebo of 26 and 53%, respectively, when applied with brushing and of 27 and 57%, respectively, when applied as slurry [29]. A 55% increase in protection was reported after using a 5,000 ppm fluoride formulation vs. a 1,450 ppm fluoride product [30]. In other *in situ* experiments, no significant erosion-and abrasion-protective effect of fluoride toothpastes was determined [36,37]. Moreover, erosive lesions are common, depite the widespread use of fluoride toothpastes. Therefore, widespread interest exists in substances other than fluoride that may increase dental hard tissue resistance to erosion.

Interventional effectiveness appears to depend not only on the dentifrice formulation and application mode, but also on the erosion model used [38]. Exposure to saliva and some dietary products can support remineralization [39]. The postulated mechanism for this effect is that the deposition of salivary calcium and phosphate onto the softened tooth surface once the erosive agent is neutralized will cause re-hardening of the enamel [38]. In an *ex vivo* study using citric acid erosion, immersion of the samples in artificial saliva caused partial re-hardening after 1–4 h and complete remineralization after 6–24 h [38]. In another study, tooth samples underwent acid erosion with grapefruit juice for 20 min followed by remineralization using Casein Phosphopeptide-Amorphous Calcium Phosphate Paste (CPP-ACP). SEM images of the samples suggested a remineralization-supportive effect by this dentifrice formulation [40]. CPP-ACP contains inorganic components which can potentially act as remineralizing agents on the enamel [41,42]. Indeed, a wide range of studies involving a plethora of toothpastes have reported varying degrees of remineralizing efficacy for many calcium and/or phosphate and/or fluoride-containing formulations [21,43].

In this study, erosive effects on the enamel surface of tooth chips were similar during use of a control or test dental gel. The SEM images of samples from each dental gel treatment group are similar, showing the faint undulating appearance of the enamel prisms and isolated circumscribed patches of surface enamel deficiencies. A few areas of typical erosive damage are visible, paralleling the results of other studies investigating enamel erosion followed by remineralization [27,28]. Based on the data from this study, it seems reasonable to conclude that the formulation’s ingredients, including the activated edathamil used for its anti-plaque effect, did not appear to adversely affect the enamel surface in its response to or recovery from an erosive challenge. However, because SEM is unable to quantify actual mineralization changes, further studies using more quantitative techniques such as microhardness, nano-hardness, or micro-chemical analysis, are necessary to solidify knowledge about the influence of the test dental gel on enamel re- and demineralization. Optimally, the dentifrice should also be tested in a longitudinal clinical study, simulating actual day to day use by patients.

## Conclusion

Using an *in vivo* model in tooth samples subjected to erosive challenge, the enamel surface appeared similar after usage of either a test dental gel (Livionex dental gel^R^) or a commonly used control gel (Colgate Total^R^). Minimal signs of residual microstructural erosion were apparent in the SEM images of samples from both groups. Based on the imaging data, the novel formulation did not adversely affect the enamel surface in its response to or recovery from an erosive challenge. Since this novel dentifrice contains no triclosan, detergents or abrasives it avoids the risk of bacterial resistance, may reduce physiological intolerance and potentially limits abrasion. Thus it may potentially represent an important addition to the currently available spectrum of dentifrices.
